# Repeated evolution of reduced visual investment at the onset of ecological speciation in high-altitude *Heliconius* butterflies

**DOI:** 10.1093/evlett/qraf017

**Published:** 2025-07-09

**Authors:** David F Rivas-Sánchez, Jake Morris, Camilo Salazar, Carolina Pardo-Díaz, Richard M Merrill, Stephen H Montgomery

**Affiliations:** School of Biological Sciences, University of Bristol, 24 Tyndall Avenue, BS8 1TQ, Bristol, United Kingdom; Division of Evolutionary Biology, Ludwig-Maximilians-Universität München, Grosshaderner Strasse 2, 82152, Planegg-Martinsried, Germany; School of Biological Sciences, University of Bristol, 24 Tyndall Avenue, BS8 1TQ, Bristol, United Kingdom; Department of Biology, Faculty of Natural Sciences, Universidad del Rosario, Carrera 24 #63c - 69, 111711, Bogotá, Colombia; Department of Biology, Faculty of Natural Sciences, Universidad del Rosario, Carrera 24 #63c - 69, 111711, Bogotá, Colombia; Division of Evolutionary Biology, Ludwig-Maximilians-Universität München, Grosshaderner Strasse 2, 82152, Planegg-Martinsried, Germany; School of Biological Sciences, University of Bristol, 24 Tyndall Avenue, BS8 1TQ, Bristol, United Kingdom

**Keywords:** neuroecology, parallel adaptations, optic lobe, antennal lobe, ecological speciation, brain plasticity

## Abstract

Colonization of new habitats is typically followed by divergent selection acting on traits that are immediately important for fitness. For example, differences between sensory environments are often associated with variation in sensory traits critical for navigation and foraging. However, the extent to which the initial response to novel sensory conditions is mediated by phenotypic plasticity, and the contribution of sensory or neural adaptation to early species divergence remains unclear. We took advantage of repeated cases of speciation in *Heliconius* butterflies with independent allopatric distributions in the west of the Colombian and Ecuadorian Andes. Using volumetric brain measurements, we analyzed patterns of investment in primary sensory processing areas of the brain across different localities and habitats. We find that a higher altitude species, *Heliconius chestertonii*, differs in levels of investment in visual and olfactory brain components compared with its lower altitude relative *H. erato venus*, mainly attributable to broad-sense heritable variation as inferred from comparisons between wild and common-garden-reared individuals. We provide evidence that this variation is consistent with divergent selection, and compare these shifts with those reported for another high-altitude species, *H. himera*, and its parapatric lowland counterpart, *H. erato cyrbia*, to demonstrate parallel reductions in the size of specific optic lobe neuropils. Conversely, for the antennal lobe, we detected different trait shifts in *H. himera* and *H. chestertonii* relative to their lowland *H. erato* neighbors. Overall, our findings add weight to the adaptive potential of neuroanatomical divergence related to sensory processing during early species formation.

## Introduction

Local adaptation may promote the evolution of reproductive isolation between populations exposed to different environments due to selection against maladapted migrants or hybrid offspring (Coyne & Orr, 2004; Nosil et al., 2005; Rundle & Nosil, 2005; Schluter, 2009). Traits important for habitat use and resource acquisition are likely exposed to divergent selection following habitat colonization (Via, 2009). In particular, because navigation and foraging require the brain to constantly interpret and respond to external cues, selection on the sensory and neural traits that underlie perception and behavior is expected to closely track changes in habitat properties such as food distribution or light regime (Dell'Aglio et al., 2024; Wcislo, 1989). Classic examples of this process largely stem from aquatic systems. For example, the brain structure of (blind) Mexican cavefish (*Astyanax mexicanus*) differs from that of their surface-dwelling counterparts, (Loomis et al., 2019), and divergence in the visual system of sympatric *Pundamilia* cichlids contributes to lower fitness when reciprocally transplanted to non-native light environments (Maan et al., 2017).

Which traits facilitate ecologically driven divergence remains unclear, but comparisons between taxa at the earlier stages of species formation may offer detailed insights into this question. This is because variation in such traits might be expected to arise first and its source can therefore be more easily discerned in closely related populations (Gonda et al., 2013). In particular, populations living in similar ecological conditions, and which have evolved similar trait shifts may reveal the effects of shared selective pressures, providing comparative evidence of adaptive divergence as opposed to neutral drift or phenotypic plasticity (Langerhans & DeWitt, 2004; Losos et al., 1998; Schluter, 2000). For example, different freshwater stickleback (*Gasterosteus aculeatus*) populations have independently lost armor plates relative to marine conspecifics (Colosimo et al., 2005), and parallel divergence in host-plant use has been reported in stick insects (*Timema cristinae*) (Nosil et al., 2002). Both cases suggest habitat shifts correlated with divergent adaptations that arise repeatedly under similar selection regimes.

Butterflies occur across a broad range of ecological conditions and provide an excellent opportunity to explore how the sensory and behavioral requirements of contrasting habitats are met by adaptations in peripheral and higher order brain structures (Couto et al., 2020). Here, we use closely related species from within the Neotropical *Heliconius erato* complex to explore how neuroanatomical trait shifts may be associated with changes in habitat. Within this group, *H. chestertonii* and *H. himera* are genetically distinct species (Van Belleghem et al., 2021) notable for their specialization to higher elevation forests in the west of the Colombian and Ecuadorian Andes, respectively (Arias et al., 2008; Jiggins et al., 1996). At their lower altitudinal boundaries, these species are in contact with lowland *H. erato* populations, with which they form narrow hybrid zones (*H. chestertonii* with *H. erato venus*, and *H. himera* with *H. erato cyrbia*) (Arias et al., 2008; Jiggins et al., 1996), providing extant proxies of the populations ancestral to *H. chestertonii* and *H. himera*. In each case, the proportion of hybrids is below that expected under random mating, despite only limited hybrid inviability (McMillan et al., 1997; Muñoz et al., 2010). This is consistent with persistent gene flow countered by the combined effects of assortative mating and frequency-dependent selection on warning color patterns (McMillan et al., 1997; Mallet et al., 1998; Merrill et al., 2014; Muñoz et al., 2010). Selection is additionally expected to relate to abrupt environmental shifts between the dense, warm, wet, and climatically stable forests inhabited by lowland *H. erato* populations, and the open, drier, and more climatically fluctuating high-elevation forests where *H. chestertonii* and *H. himera* are distributed (Arias et al., 2008; Jiggins et al., 1996).

Lower elevation *H. e. cyrbia* and higher elevation *H. himera*, have diverged in traits related to physiology, life history, development, and flight behavior (Davison et al., 1999; Dell'Aglio et al., 2022). This is closely mirrored in the ecologically equivalent species pair *H. e. venus*–*H. chestertonii*, implying independent, parallel adaptations to high altitude (Rivas-Sánchez et al., 2023, 2024). *H. e. cyrbia* and *H. himera* also exhibit distinct neuroanatomies that result in greater relative tissue investment in the optic lobe of *H. e. cyrbia* and the antennal lobe of *H. himera* (Montgomery & Merrill, 2017). These differences are heritable and have been interpreted as divergent adaptations shaped by the sensory conditions of their respective forest environments (Montgomery & Merrill, 2017). This conclusion is supported by behavioral data showing that contrasting sensory investment predicts differential weighting of information from visual and olfactory sensory modalities during foraging (Dell'Aglio et al., 2022). Additional evidence of the adaptive significance of these shifts could be provided if they are replicated in a separate, ecologically similar divergence event.

We predicted that *H. chestertonii*, a species genetically independent (Van Belleghem et al., 2021) but ecologically equivalent to *H. himera* ( Rivas-Sánchez et al., 2023, 2024), would exhibit neuroanatomical trait shifts relative to lowland *H. e. venus* similar to those observed between *H. e. cyrbia* and *H. himera*. This is expected if divergent ecological selection from wet to dry forests is met with adaptations in brain structure in derived high-altitude conditions. To test this, we drew comparisons using volumetric data obtained from wild-caught and insectary-reared *H. e. venus* and *H. chestertonii*. We then combined our data with previously published brain structure measurements of *H. e. cyrbia* and *H. himera* (Montgomery & Merrill, 2017) to formally test for parallel neural evolution associated with shifts to high-altitude forests.

## Methods

### Collections in the field and in common garden

We collected wild *H. e. venus* (Σ*n* = 20, males = 13, females = 7) and *H. chestertonii* (Σ*n* = 18, males = 12, females = 6) using hand nets in forests located in southwest Colombia at low (Buenaventura, N03°50′0.04′′ W77°15′45.1′′ and La Barra, N03°57.558420 W77°22.705080, 0–22 m above sea level) and high altitude (Montañitas N03°40′36.3′′ W76°31′19.4′′ and El Saladito N03°29′11.5′′ W76°36′39.8′′, 1,746–1,812 m above sea level) in January 2020 and January 2021. Because these sites are distant from the contact zone and hybrids have never been reported in these areas, individuals are considered “faithful” representatives of their species. To explore potential habitat effects on brain structure, we also included first-generation insectary-reared *H. e. venus* (Σ*n* = 18, males = 5, females = 13) and *H. chestertonii* (Σ*n* = 17, males = 7, females = 10) bred from independent wild-caught females. These butterflies were reared in the José Celestino Mutis research station in La Vega (1,485 m), Colombia and sampled after having reached maturity (∼10 days), when posteclosion brain growth in *Heliconius* plateaus (Montgomery et al., 2016). To test for parallel trait shifts in high-altitude populations, we additionally incorporated volumetric brain measurements of wild-caught *H. e. cyrbia* (Σ*n* = 16, males = 8, females = 8) and *H. himera* (Σ*n* = 16, males = 8, females = 8), obtained from Montgomery & Merrill (2017).

### Brain tissue preparation

We dissected, fixed, and prepared whole brains for indirect immunofluorescence staining against synapsin, a protein expressed in presynaptic sites (anti-SYNORF1; Developmental Studies Hybridoma Bank, Department of Biological Sciences, University of Iowa, IA; RRID:AB_2315424), with a Cy2-conjugated affinity-purified polyclonal goat antimouse IgG (H + L) secondary antibody (Stratech Scientific, Suffolk, UK; Jackson ImmunoResearch Cat No. 115-225-146, RRID:AB_2307343). This protocol reveals the borders of neuropils, synapse dense brain regions that reflect functionally informative divisions in insect brains (Ott, 2008).

### Confocal microscope imaging and volumetric reconstructions

**Figure 1.**
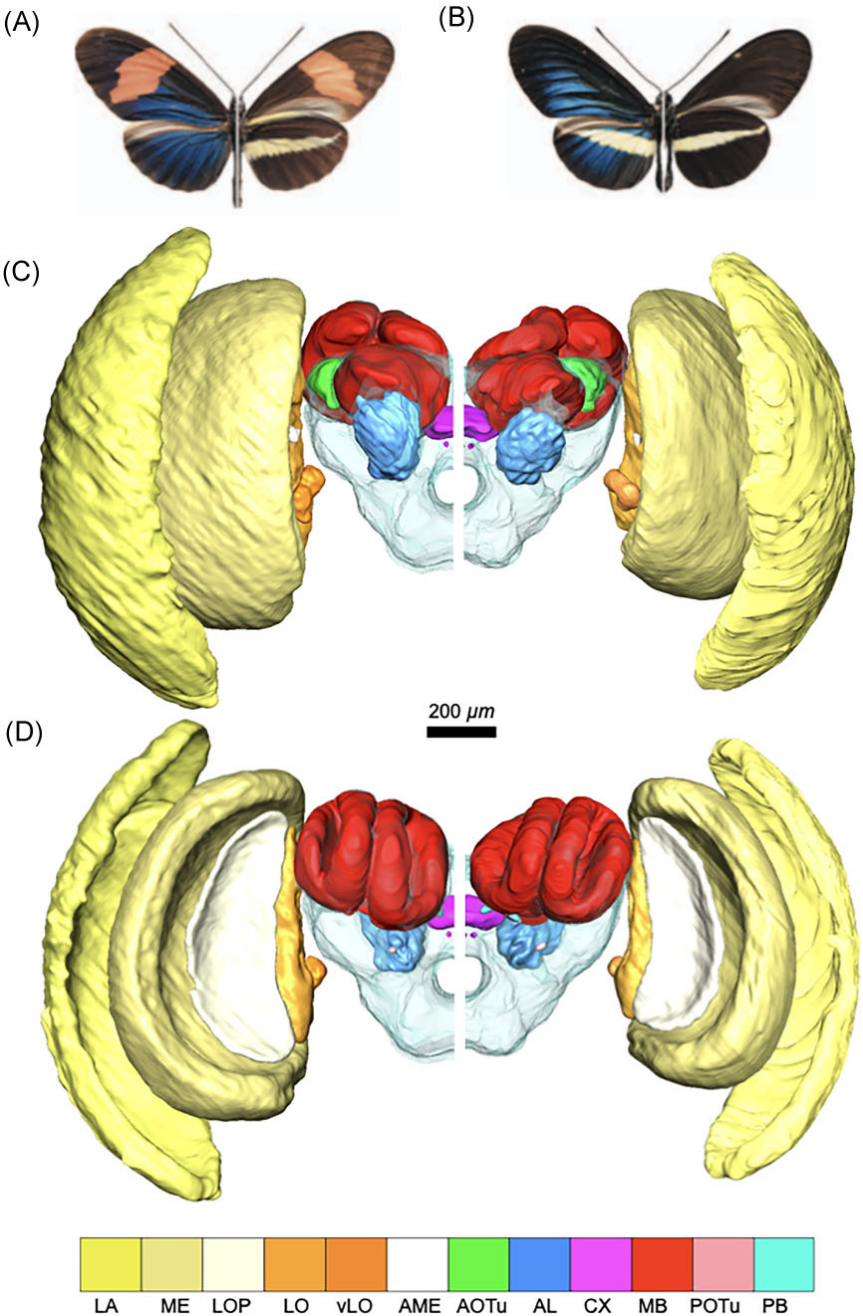
Upper row. Dorsal (left side) and ventral (right side) overview of the wing color patterns of (A) *H. e. venus* and (B) *H. chestertonii*. Middle and bottom rows. Rostral (C) and caudal (D) view of volumetric reconstructions of the brains of wild male *H. e. venus* (left half) and *H. chestertonii* (right half). The brain images showcase the undifferentiated regions of the central brain (transparent), the lamina (LA), the medulla (ME), the lobula plate (LOP), the lobula (LO), the ventral lobula (vLO), the accessory medulla (AME), the anterior optic tubercle (AOTu), the posterior optic tubercle (POTu), the mushroom bodies (MB), the protocerebral bridge (PB), the central complex (CX), and the antennal lobe (AL).

We performed imaging on a confocal laser-scanning microscope (Leica TCS SP5 or SP8; Leica Microsystem) with a 10× dry objective (numerical aperture 0.4) to produce 512 × 512 pixels *x*–*y* resolution image stacks with a 2 μm *z*-step. We used Amira 5.5 (Thermo-Fisher Scientific) to merge image stacks and assign voxels to neuropils of interest. This allowed us to reconstruct and obtain volumetric measurements of sensory neuropils in the optic lobe (lamina, medulla, lobula plate, lobula, ventral lobula, lobula, and accessory medulla) and the central brain (antennal lobes, anterior optic tubercle [AOTu], and posterior optic tubercle [POTu]). Following Montgomery & Merrill (2017), we measured the total volume of the central brain and subtracted the volume of the antennal lobes, the AOTu, the POTu, the mushroom bodies, the protocerebral bridges, and central complex to obtain the volume of the undifferentiated regions of the central brain (rCBRs), which was used as an allometric control (i.e., independent measure of body size) (Figure 1). Importantly, rCBR does not vary significantly between sexes (*n* = 38, *F* = 0.143, *P* = 0.706), species (*n* = 38, *F* = 0.000, *P* = 0.992), or with habitat type (*n* = 67, *F* = 0.023, *P* = 0.879).

### Statistical analyses

To support previous interpretations of the *erato* complex being an ancestrally low-altitude range, with the higher altitudinal ranges of *H. himera* and *H. chestertonii*’s therefore being derived, we performed an ancestral state reconstruction using altitudinal data on extant Heliconiini species (Supplementary Material). For trait analysis within our species pairs, we used linear mixed-effects models (lmer function in the lmer package in R [Bates et al., 2015]) to assess the scaling relationships between the neuropils of interest and rCBR. In our models, the volume of each neuropil was a function of the fixed factors “rCBR” and “species,” as well as the random factor “sex.” The effect of “species” was determined by comparing model performance with and without the term using Wald *Χ*^2^ tests (Anova function in the R package CAR [Fox & Weisberg, 2018]).

We next implemented standardized major axis regression analyses (SMATR) to confirm nonallometric shifts in brain structure across wild-caught *H. e. venus* and *H. chestertonii* (SMATR package in R [Warton et al., 2012]). These analyses test for significant between-groups shifts in the allometric relationship log *y* = βlog *x* + α, which describes the association between a given trait (*y*, i.e., volume of neuropil of interest) and an independent measure of size or allometric control (*x*, i.e., volume of rCBR). β is the slope and α is the elevation of the line of fit. Shifts in slope reflect different scaling relationships between the species being compared, whereas changes in the elevation (grade shifts) are interpreted as nonallometric divergence (Kruska, 2005). Both may result from individual brain regions responding to selection independently of other structures or overall brain size, known as mosaic brain evolution (Barton & Harvey, 2000). Major axis shifts occur along a shared allometric relationship and underly changes in neuropil volume in concert with the rest of the brain (Barton & Harvey, 2000; Montgomery et al., 2016).

We also performed these tests separately in individuals reared in common-garden insectary conditions to determine the extent to which between-species patterns are attributable to broad-sense heritable variation in brain structure. We additionally estimated phenotypic differentiation (*P*_st_) for sensory neuropils between insectary *H. e. venus* and *H. chestertonii* (Pst function in the *Pstat* R package [Da Silva & Da Silva, 2018; allometric correction *Res*) and compared it with a genome-wide *F*_st_ distribution (41,677,795 SNPs across chromosomes 4–9, 11–14, 16, 17, and 20, as they do not contain non-neutral divergence peaks between *H. erato* subspecies, following Van Belleghem et al. [2017]). *P*_st_ is an analogue to *F*_st_ that reflects between-population phenotypic divergence (Spitze, 1993). Differentiation is greater than expected from neutral evolution if *P*_st_ exceeds *F*_st_ (Da Silva & Da Silva, 2018). Because *P*_st_ is sensitive to assumptions of the proportion of phenotypic variance attributable to genetic effects between (*c*) and within populations (*h*^2^) populations (Brommer, 2011), we estimated it across different quantitative genetic scenarios (*c* = 1, expected in common garden; *h*^2^ = 0.5–1).

We additionally created a combined dataset with volumetric brain measurements of *H. e. cyrbia, H. himera, H. e. venus*, and *H. chestertonii* to test for shared patterns of ecologically driven divergence. The habitat of each species has been previously designated as low (*H. e. cyrbia* and *H. e. venus*) or high altitude (*H. himera* and *H. chestertonii*) based on patterns of macroclimatic variation across their distribution (Rivas-Sánchez et al., 2023). We used linear mixed models where variation in neuropil volume was partitioned in that attributable to the fixed factors “habitat type” (low or high altitude), “locality” (Colombia or Ecuador), and their interaction. Comparing models with and without these terms allowed detecting significant effects on the volume of specific neuropils stemming from shared sources of ecologically based selection (“habitat type”), unique histories of each divergence event (“locality”), and unique evolutionary responses to shared habitat shifts (“interaction”) (Langerhans & DeWitt, 2004). These models included our allometric control (“rCBR”) as a fixed factor and “sex” as random term. The relative contribution of each factor to trait variation was subsequently estimated using semipartial *R*^2^ measures using the *r2Beta* function (Kenward Roger method in the R package *r2glmm* [Jaeger et al., 2017]). Brain data, detailed test results, and annotated R scripts used here are provided in the Supplementary Materials (Supplementary Tables S1 and S2).

## Results

### Heritable divergence in sensory brain structures between *H. e. venus* and *H. chestertonii*

**Figure 2.**
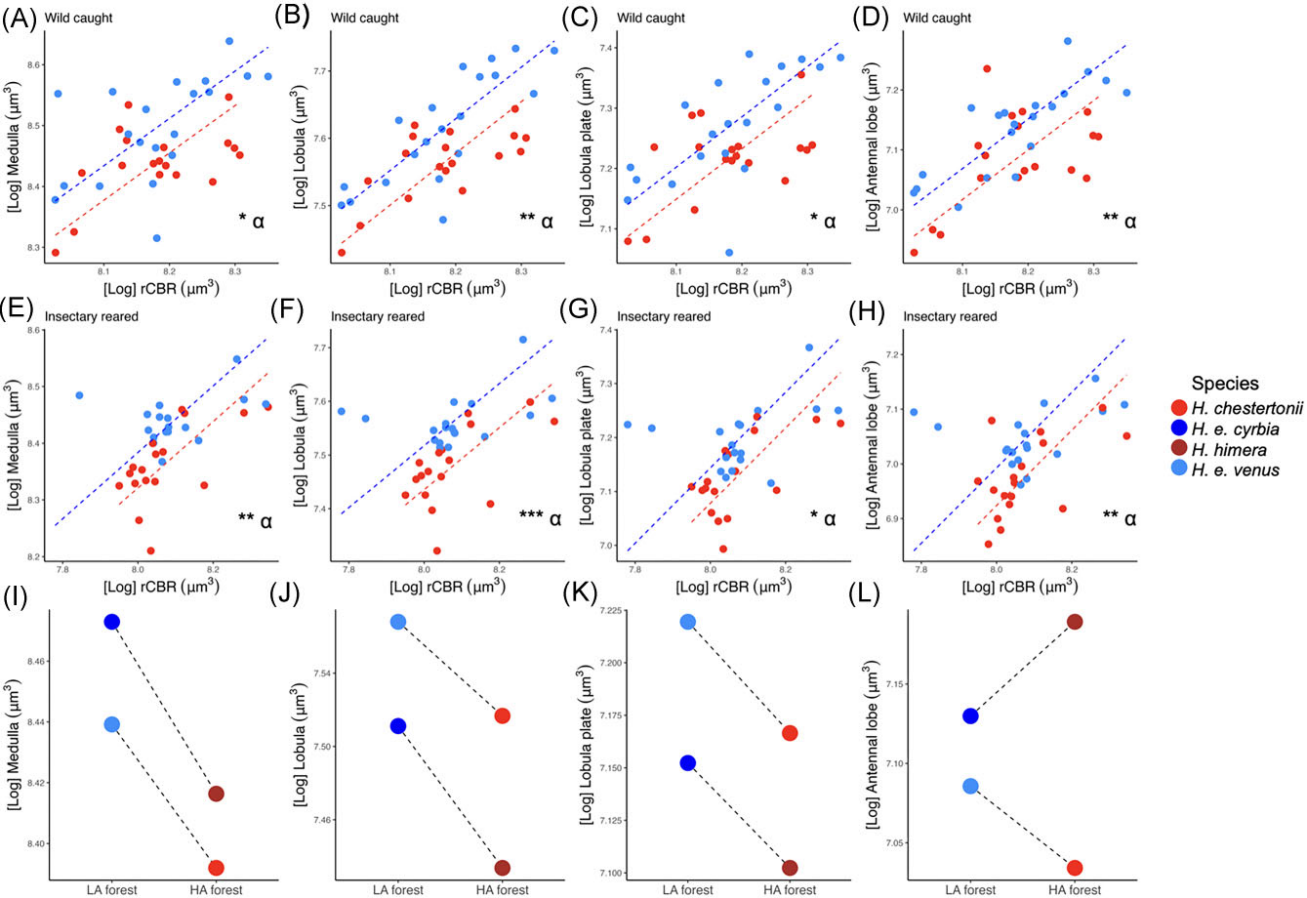
First row. Grade (⍺) shifts between wild *H. chestertonii* and *H. e venus* in scaling relationships against the rCBR of the (A) medulla, (B) lobula, (C) lobula plate, and (D) antennal lobe. Second row. Grade (⍺) shifts between insectary-reared *H. chestertonii* and *H. e venus* in the scaling relationships against the rCBR of the (E) medulla, (F) lobula, (G) lobula plate, and (H) antennal lobe. For all the plots, points and dashed lines represent, respectively, individuals and allometries between neuropil and rCBR volume for *H. chestertonii* (red) and *H. e. venus* (blue). Asterisks convey significance levels of Grade (⍺) shifts (Wald *Χ*^2^ statistic). * *p* < 0.05; ** *p* < 0.01; *** *p* < 0.001. Third row. Levels of parallelism in *Heliconius* species pairs in the relative volume of the (I) medulla, (J) lobula, (K) lobula plate, and (L) antennal lobe. Colored dots convey the volume of neuropils estimated with the allometric function log *y* = βlog *x* + α for butterflies with an rCBR size of 8.128 (average [Log] rCBR (µm^3^) of wild-caught butterflies, Supplementary Table S8). Dashed lines connect species from the same locality and different forest types. In all the plots, *H. chestertonii* is in red, *H. e. venus* in blue, *H. himera* in firebrick, and *H. e. cyrbia* in dodger blue. LA = low-altitude forest and HA = high-altitude forest.

Model comparisons revealed differences between wild *H. e. venus* and *H. chestertonii* in the volume of the medulla (*n* = 38, *Χ*_1_^2^ = 8.335, *p* < 0.05), the lobula (*n* = 38, *Χ*_1_^2^ = 11.590, *p* < 0.01), the lobula plate (*n* = 38, *Χ*_1_^2^ = 7.003, *p* < 0.05), and the antennal lobe (*n* = 38, *Χ*_1_^2^ = 7.499, *p* < 0.01), which are generally larger in *H. e. venus* compared with *H. chestertonii*. SMATR analyses confirmed that this variation is driven by nonallometric grade shifts (Figure 2A–D and Supplementary Table S3) affecting specific optic lobe neuropils (medulla, Wald *Χ*_1_^2^ = 5.454, *p* < 0.05; lobula, Wald *Χ*_1_^2^ = 8.469, *p* < 0.05; and lobula plate, Wald *Χ*_1_^2^ = 5.459, *p* < 0.05) and the antennal lobe (Wald *Χ*_1_^2^ = 7.225, *p* < 0.05).

This pattern was preserved in our sample of insectary-reared individuals (grade shifts in the medulla, Wald *Χ*_1_^2^ = 9.328, *p* < 0.001; lobula, Wald *Χ*_1_^2^ = 11.77, *p* < 0.001; the lobula plate, Wald *Χ*_1_^2^ = 5.945, *p* < 0.05; and antennal lobe, Wald *Χ*_1_^2^ = 7.000, *p* < 0.05), which in addition, exhibited a between-species slope shift in the lamina (LR_1_^2^ = 4.369, *p* < 0.05) and major-axis shifts in the ventral lobula (Wald *Χ*_1_^2^ = 3.886, *p* < 0.05) and the AOTu (Wald *Χ*_1_^2^ = 4.306, *p* < 0.05) (Figure 2E–H and Supplementary Table S4). Further comparisons showed that variation between insectary-reared and wild *H. chestertonii* are mostly explained by allometric scaling, with no significant β or α shifts except for the POTu (Supplementary Table S5). Allometric scaling also explains differences between *H. e. venus* of different origins (wild vs. insectary reared) except for the medulla, the lobula, and the lamina, which showed slope shifts between insectary and wild conspecifics (Supplementary Table S6). Across a broad range of quantitative genetic scenarios (varying *h*^2^), the lobula *P*_st_ estimate significantly exceeds neutral evolution expectations between insectary *H. e. venus* and *H. chestertonii* (e.g., *P*_st_: 0.915, *p* < 0.05, *h*^2^ = 1). Other neuropils are more sensitive to *h*^2^ values but, under reasonable estimates of *h*^2^ consistent with our common-garden experiments, *P*_st_ also exceeds *F*_st_ for the medulla (*P*_st_: 0.912, *p* < 0.05, *h*^2^ < 0.80), the antennal lobe (*P*_st_: 0.915, *p* < 0.05, *h*^2^ < 0.66), the lobula plate (*P*_st_: 0.913, *p* < 0.05, *h*^2^ < 0.57), and the AOTu (*P*_st_: 0.915, *p* < 0.05, *h*^2^ < 0.50) (Supplementary Table S7).

### Consistent shifts in visual investment during repeated, independent colonization of high-altitude habitats

Our ancestral state reconstruction supports the inference that the last common ancestor of the *erato* complex had a low-altitudinal range similar to all extant *H. erato* populations, excluding *H. himera* and *H. chestertonii* (Supplementary Material). For the brain regions identified as divergent in wild-caught individuals, we compared linear mixed models explaining variation in neuropil size across *H. e. venus, H. chestertonii, H. e. cyrbia*, and *H. himera*. We found significant “habitat type” effects on the medulla (*Χ*_1_^2^ = 21.111, *p* < 0.001, semipartial *R*^2^ = 0.245), the lobula (*Χ*_1_^2^ = 30.905, *p* < 0.001, *R*^2^ = 0.322), and the lobula plate (*Χ*_1_^2^ = 13.911, *p* < 0.001, *R*^2^ = 0.174), which, for a given rCBR size, are significantly smaller in the high-altitude specialists *H. chestertonii* and *H. himera* than in their parapatric lowland *H. erato* populations (Figure 2I–K). We also identified significant “locality” effects on the lobula (*Χ*_1_^2^ = 67.665, *p* < 0.001) and the lobula plate (*Χ*_1_^2^ = 31.954, *p* < 0.001). In addition, we found a significant effect of the habitat type–locality interaction on the antennal lobe (*Χ*_1_^2^ = 15.4913, *p* < 0.001), consistent with contrasting habitat-type effects in its volume across Colombia and Ecuador (Figure 2L).

## Discussion

We leveraged replicated, relatively recent speciation events in *Heliconius* to test for adaptive shifts in neuroanatomy associated with ecological divergence across forest types. First, we found that wild *H. chestertonii* individuals sampled from their high-altitude forest habitats in Colombia show nonallometric reductions in the size of specific optic lobe neuropils compared with *H. e. venus* butterflies captured in lower altitude forests. Next, by rearing these same species under common-garden conditions, we determined that broad-sense heritable variation largely underlies this pattern of divergence. Next, we observed that population differences in the optic lobe exceed that expected from neutral evolution. In addition, the results of our analyses incorporating data on wild *H. e. cyrbia* and *H. himera* demonstrate that optic lobe size variation is at least partially attributable to shared selection regimes across the geographically separated high-altitude forests inhabited by *H. himera* and *H. chestertonii*, likely related to parallel adaptation in similar visual environments. In contrast, the antennal lobe shows disparate trait shifts across *H. himera* and *H. chestertonii*, suggesting different responses to ostensibly equivalent habitat shifts, and independence between brain regions associated with separate sensory modalities.

Conserved scaling relationships between brain components are thought to reflect the effects of stabilizing selection limiting the independent evolution of functionally linked brain regions (Montgomery et al., 2016). Mosaic-like patterns of divergence in brain structure, on the other hand, suggest selection targeting specific brain regions resulting in adaptive behavioral shifts (Barton & Harvey, 2000). We detected significant levels of between-species variation in the size of multiple sensory neuropils in wild *H. e. venus* and *H. chestertonii*. Allometric analyses further showed that these differences consist of grade shifts that affect the medulla, the lobula, the lobula plate, and the antennal lobe, resulting in those neuropils being functionally smaller in wild *H. chestertonii* relative to *H. e. venus* for a given brain size. Equivalent grade shifts were detected in the same neuropils of butterflies reared in common-garden conditions, where altitude and climatic conditions were similar to those naturally experienced by *H. chestertonii* (Rivas-Sánchez et al., 2023). Thus, it is likely that the patterns of brain divergence we observed in wild individuals represent broad-sense heritable variation rather than environmentally induced responses. A slope shift was detected for the lamina in insectary (but not wild) butterflies, resulting in this neuropil being smaller in common-garden *H. e. venus* at higher values of overall brain size. This could represent a nonadaptive plastic response prompted in a high-altitude environment (Ghalambor et al., 2007), but this hypothesis requires further experimental work to test.

Insectary-reared *H. chestertonii* exhibited major axis shifts in nearly all measured neuropils compared with wild-caught conspecifics, which can be explained by concerted reductions in overall brain size, perhaps due to contrasting experiences between forests and insectary cages (Montgomery et al., 2016). Interestingly, although an equivalent comparison in *H. e. venus* revealed similar overall brain size reductions in insectary-reared butterflies, this group also showed nonallometric shifts relative to wild conspecifics that bring it closer to a *H. chestertonii*-like phenotype (i.e., slope shifts reducing the volume of the medulla and the lobula compared with wild conspecifics at higher values of overall brain size). A comprehensive comparison between *H. e. venus* siblings exposed to derived high- and ancestral low-altitude habitats would help determine if neural plasticity acts as a “pacemaker” mechanism facilitating nonallometric reductions in these sensory neuropils following exposure to high-altitude conditions (Levis & Pfennig, 2016). In insectary butterflies, between-species *P*_st_ significantly exceeds *F*_st_ for the lobula when phenotypic variation between individuals is assumed to be exclusively caused by additive genetic effects. If potential additional factors are considered (e.g., epistasis and dominance, entailing *h*^2^ < 1), between-species differences in the medulla and the lobula plate are also consistent with divergent selection differentiating *H. e. venus* and *H. chestertonii*. Notably, covariation between the medulla and lobula is weak (Supplementary Table S9), suggesting selection independently targets optic lobe components.

In the independent high-altitude specialist *H. himera*, multiple optic lobe neuropils are reduced compared with the same structures in *H. e. cyrbia* from lower altitude (Montgomery & Merrill, 2017). Our results show a parallel pattern of variation in three optic lobe neuropils in *H. e. venus* and *H. chestertonii*, which are segregated in ecologically similar forest types at low and high altitude (Rivas-Sánchez et al., 2023, 2024). These include the medulla, which in *Drosophila* and honeybees has been implicated in light parallelization, color vision, and motion detection (Borst, 2009; Morante & Desplan, 2004; Paulk et al., 2009), as well as the lobula and the lobula plate, which are involved in escape/chase reactions to visual stimuli and motion computation, respectively (Douglass & Strausfeld, 1998; Hausen, 1984). Our model comparisons incorporating data from the four species favor divergent selection as a factor explaining these repeated trends of optic lobe size reduction across the Ecuador and Colombian localities. In particular, “habitat type” explains 17%–32% of the overall neuropil size variation in these species, implying independent, repeated trait shifts, consistent with adaptation shaped by similar ecological conditions (Bolnick et al., 2018; Langerhans & DeWitt, 2004). This reflects patterns seen in the more distantly related species pair *H. cydno*–*H. melpomene*, where habitat partitioning along gradients of increasing light intensity in forest interior-forest edge axes is accompanied by non-neutral reductions in the size of the optic lobe and the AOTu, as well as lower number of eye facets and reduced corneal area (Montgomery et al., 2021; Seymoure et al., 2015; Wright et al., 2023).

Perceiving and processing sensory information is energetically expensive (Laughlin et al., 1998) and these high metabolic demands result in selection for efficiency, ultimately regulating sensory system design (Laughlin, 2001; Laughlin et al., 1998; Niven & Laughlin, 2008). For example, Strepsirrhine primates that experience seasonal low food availability have smaller brains, thought to be due to an overall reduced energetic budget (van Woerden et al., 2010). Sensory environments can also influence how such costs are offset. In *Drosophila* populations reared in captivity, selection for finding resources using visual cues is relaxed relative to natural conditions while selective pressures for low energy consumption are maintained, leading to the evolution of smaller (i.e., less energetically demanding) eyes over few generations (Tan et al., 2005). A similar pattern to ours is also observed in birds, which exhibit smaller eye size in association with brighter, open habitats (Liu et al., 2023). Previous studies have shown that low-altitude forests in Colombia and Ecuador have denser vegetation, taller canopies, and are subject to lower levels of solar radiation, (Dell'Aglio et al., 2022; Rivas-Sánchez et al., 2023, 2024). In visually guided diurnal butterflies, such as *Heliconius* (Dell'Aglio et al., 2016; Hausmann et al., 2021; Yuan et al., 2010), these dimmer conditions may therefore entail more visually complex environments compared with high-altitude forests. Thus, shifts toward a reduced volume in some optic lobe neuropils in *H. chestertonii* and *H. himera* could potentially result from sustained selection for low energetic costs concurrent with reduced requirements on visual performance in brighter, high-altitude habitats. The frequency of encounters between locally adapted individuals and immigrants may be lowered if survival in immigrants is decreased due to a mismatch to the alternative sensory environment (Maan et al., 2017). This “immigrant inviability” may represent an important reproductive barrier contributing to speciation (Coyne & Orr, 2004; Ingley et al., 2016; Nosil et al., 2005; Prada & Hellberg, 2014). In addition, in *Heliconius* hybrids, the scaling relationships and gene expression of optic lobe neuropils may be intermediate or mismatched, relative to parental species, potentially reducing individual fitness (Arias et al., 2008; Jiggins & Mallet, 2000; Montgomery et al., 2021). We hypothesise that these factors may therefore constitute pre and postzygotic barriers to gene flow that contribute to early species divergence in absence of geographic isolation between the low- and high-altitude habitats.

In contrast to consistent changes in the visual neuropils, we identified incongruent evolutionary responses in the antennal lobe between Colombian and Ecuadorian localities. Among Lepidoptera, variation in the relative size of the antennal lobe is assumed to reflect a different emphasis on olfactory cues between species in habitats with contrasting availability of visual information (Montgomery & Ott, 2015; Morris et al., 2021; Stöckl et al., 2016). While investment in this neuropil is higher in *H. himera* in respect to *H. e. cyrbia* (Montgomery & Merrill, 2017), here, we show that it is lower in *H. chestertonii* compared with its lowland counterpart *H. e. venus*, entailing locality specific responses to habitat shifts, and independent evolution of brain components linked to vision and olfaction, without apparent developmental trade-offs in investment in sensory modalities (Farnworth & Montgomery, 2022). Variation in the antennal lobe is heritable (broad sense) in *H. e. venus* and *H. chestertonii*, but not in *H. e. cyrbia* and *H. himera* (Montgomery & Merrill, 2017). Therefore, a possibility is that the evolution of this neuropil is influenced by lasting effects of recent gene flow with other *Heliconius* species or genetic drift (Moore & Hendry, 2005; Oke et al., 2017; Stuart et al., 2017). Alternatively, the habitat categorizations employed here might not capture aspects of environmental variation that may exert locality specific selective pressures (Bolnick et al., 2018; Stuart et al., 2017) on the antennal lobe across the habitats of *H. himera* and *H. chestertonii*. For instance, relative humidity and rainfall are significantly higher in the habitat of *H. chestertonii* compared with the forests inhabited by *H. himera* (Rivas-Sánchez et al., 2024). Further research could explore whether such differences affect the sensory background under which olfactory cues are perceived, which may in turn relate to the patterns of antennal lobe variation we observed.

In summary, our study presents evidence of broad-sense heritable neuroanatomical divergence between two closely related species from the *H. erato* complex that are segregated in forests at different altitude in Colombia. Similar to physiological, life history, and behavioral traits ( Rivas-Sánchez et al., 2023, 2024), some of these changes are mirrored in a separate pair of species that occupy similar ecological niches in Ecuador. Additionally, phenotypic divergence is underlain by nonallometric grade shifts and is higher than the expected under neutrality, suggesting the action of divergent selection within each pair. Although we are limited to two naturally occurring parallel divergence events, these findings strongly implicate local adaptation in response to shared sensory environments. Habitat variation between different forest types likely results in fitness deficits in migrants and hybrids with alternative brain phenotypes, facilitating reproductive isolation. However, these evolutionary responses are not perfectly parallel, suggesting lineage specific evolutionary responses driven by cryptic sources of environmental variation.

## Supplementary Material

qraf017_Supplemental_Files

## Data Availability

Supplementary information and raw data can be found in Supplementary Tables S1–S9, annotated R scripts, and the file “Supplementary Material S1.” Files are also available in the Dryad Digital Repository (DOI: 10.5061/dryad.02v6wwqdg).

## References

[bib1] Arias C. F., Munoz A. G., Jiggins C. D., Mavarez J., Bermingham E., Linares M. (2008). A hybrid zone provides evidence for incipient ecological speciation in *Heliconius* butterflies. Molecular Ecology, 17(21), 4699–4712. 10.1111/j.1365-294X.2008.03934.x 18828780

[bib2] Barton R. A., Harvey P. H. (2000). Mosaic evolution of brain structure in mammals. Nature, 405(6790), 1055–1058. 10.1038/35016580 10890446

[bib3] Bates D., Mächler M., Bolker B., Walker S. (2015). Fitting linear mixed-effects models using lme4. Journal of Statistical Software, 67, 1–48.

[bib4] Bolnick D. I., Barrett R. D. H., Oke K. B., Rennison D. J., Stuart Y. E. (2018). (Non)Parallel evolution. In Annual review of ecology, evolution, and systematics (Vol. 49, pp. 303–330.).

[bib5] Borst A. (2009). *Drosophila*’s view on insect vision. Current Biology, 19(1), R36–R47. 10.1016/j.cub.2008.11.001 19138592

[bib6] Brommer J. E. (2011). Whither Pst? The approximation of Qst by Pst in evolutionary and conservation biology. Journal of Evolutionary Biology, 24(6), 1160–1168. 10.1111/j.1420-9101.2011.02268.x 21457173

[bib7] Colosimo P. F., Hosemann K. E., Balabhadra S., Villarreal Jr G., Dickson M., Grimwood J., Schmutz J., Myers R. M., Schluter D., Kingsley D. M. (2005). Widespread parallel evolution in sticklebacks by repeated fixation of ectodysplasin alleles [Article]. Science, 307(5717), 1928–1933. 10.1126/science.1107239 15790847

[bib8] Couto A., Wainwright J. B., Morris B. J., Montgomery S. H. (2020). Linking ecological specialisation to adaptations in butterfly brains and sensory systems. Current Opinion in Insect Science, 42, 55–60. 10.1016/j.cois.2020.09.002 32979531

[bib9] Coyne J. A., Orr H. A. (2004). Speciation. Sinauer. https://books.google.com.ec/books?id=2Y9rQgAACAAJ

[bib10] Da Silva S. B., Da Silva A. (2018). Pstat: An R package to assess population differentiation in phenotypic traits. The R Journal, 10(1), 447. 10.32614/RJ-2018-010

[bib11] Davison A., McMillan W. O., Griffin A. S., Jiggins C. D., Mallet J. L. (1999). Behavioral and physiological differences between two parapatric *Heliconius* species. Biotropica, 31(4), 661–668. 10.1111/j.1744-7429.1999.tb00415.x

[bib12] Dell'Aglio D. D., Losada M. E., Jiggins C. D. (2016). Butterfly learning and the diversification of plant leaf shape. Frontiers in Ecology and Evolution, 4, 81. 10.3389/fevo.2016.00081

[bib13] Dell'Aglio D. D., McMillan W. O., Montgomery S. H. (2022). Shifting balances in the weighting of sensory modalities are predicted by divergence in brain morphology in incipient species of *Heliconius* butterflies. Animal Behaviour, 185, 83–90. 10.1016/j.anbehav.2022.01.003

[bib14] Dell'Aglio D. D., Mena S., Mauxion R., McMillan W. O., Montgomery S. H. (2022). Divergence in *Heliconius* flight behaviour is associated with local adaptation to different forest structures. Journal of Animal Ecology, 91(4), 727–737. 10.1111/1365-2656.13675 35157315

[bib15] Dell'Aglio D. D., Rivas-Sánchez D. F., Wright D. S., Merrill R. M., Montgomery S. H. (2024). The sensory ecology of speciation. Cold Spring Harbor Perspectives in Biology, 16(1), Article a041428. 10.1101/cshperspect.a041428 38052495 PMC10759811

[bib16] Douglass J. K., Strausfeld N. J. (1998). Functionally and anatomically segregated visual pathways in the lobula complex of a calliphorid fly. Journal of Comparative Neurology, 396(1), 84–104. 10.1002/(SICI)1096-9861(19980622)396:1<84::AID-CNE7>3.0.CO;2-E 9623889

[bib17] Farnworth M. S., Montgomery S. H. (2022). Complexity of biological scaling suggests an absence of systematic trade-offs between sensory modalities in *Drosophila*. Nature Communications, 13(1), 2944. 10.1038/s41467-022-30579-y PMC9135755 35618728

[bib18] Fox J., Weisberg S. (2018). An R companion to applied regression. Sage Publications.

[bib19] Ghalambor C. K., McKay J. K., Carroll S. P., Reznick D. N. (2007). Adaptive versus non-adaptive phenotypic plasticity and the potential for contemporary adaptation in new environments. Functional Ecology, 21(3), 394–407. 10.1111/j.1365-2435.2007.01283.x

[bib20] Gonda A., Herczeg G., Merilä J. (2013). Evolutionary ecology of intraspecific brain size variation: A review. Ecology and Evolution, 3(8), 2751–2764. 10.1002/ece3.627 24567837 PMC3930043

[bib21] Hausen K. (1984). The lobula-complex of the fly: Structure, function and significance in visual behaviour. In Photoreception and vision in invertebrates (pp. 523–559.). Springer. 10.1007/978-1-4613-2743-1_15

[bib22] Hausmann A. E., Kuo C.-Y., Freire M., Rueda-M N., Linares M., Pardo-Diaz C., Salazar C., Merrill R. M. (2021). Light environment influences mating behaviours during the early stages of divergence in tropical butterflies. Proceedings of the Royal Society B: Biological Sciences, 288(1947), 20210157. 10.1098/rspb.2021.0157 PMC8059652 33757348

[bib23] Ingley S. J., Camarillo H., Willis H., Johnson J. B. (2016). Repeated evolution of local adaptation in swimming performance: population-level trade-offs between burst and endurance swimming in *Brachyrhaphis* freshwater fish [Article]. Biological Journal of the Linnean Society, 119(4), 1011–1026. 10.1111/bij.12852

[bib24] Jaeger B. C., Edwards L. J., Das K., Sen P. K. (2017). An *R*^2^ statistic for fixed effects in the generalized linear mixed model. Journal of Applied Statistics, 44(6), 1086–1105. 10.1080/02664763.2016.1193725

[bib26] Jiggins C. D., McMillan W. O., Neukirchen W., Mallet J. (1996). What can hybrid zones tell us about speciation? The case of *Heliconius erato* and *H. himera* (Lepidoptera: Nymphalidae). Biological Journal of the Linnean Society, 59(3), 221–242.

[bib25] Jiggins C. D., Mallet J. (2000). Bimodal hybrid zones and speciation. Trends in Ecology & Evolution, 15(6), 250–255. 10.1016/S0169-5347(00)01873-5 10802556

[bib27] Kruska D. C. (2005). On the evolutionary significance of encephalization in some eutherian mammals: Effects of adaptive radiation, domestication, and feralization. Brain, Behavior and Evolution, 65(2), 73–108. 10.1159/000082979 15627722

[bib28] Langerhans R. B., DeWitt T. J. (2004). Shared and unique features of evolutionary diversification. The American Naturalist, 164(3), 335–349. 10.1086/422857 15478089

[bib29] Laughlin S. B. (2001). Energy as a constraint on the coding and processing of sensory information. Current Opinion in Neurobiology, 11(4), 475–480. 10.1016/S0959-4388(00)00237-3 11502395

[bib30] Laughlin S. B., de Ruyter van Steveninck R. R., Anderson J. C. (1998). The metabolic cost of neural information. Nature Neuroscience, 1(1), 36–41. 10.1038/236 10195106

[bib31] Levis N. A., Pfennig D. W. (2016). Evaluating ‘plasticity-first’ evolution in nature: Key criteria and empirical approaches. Trends in Ecology & Evolution, 31(7), 563–574. 27067134 10.1016/j.tree.2016.03.012

[bib32] Liu Y., Jiang Y., Xu J., Liao W. (2023). Evolution of avian eye size is associated with habitat openness, food type and brain size. Animals, 13(10), 1675. https://www.mdpi.com/2076-2615/13/10/1675 37238105 10.3390/ani13101675 PMC10215482

[bib33] Loomis C., Peuß R., Jaggard J. B., Wang Y., McKinney S. A., Raftopoulos S. C., Raftopoulos A., Whu D., Green M., McGaugh S. E., Rohner N., Keene A. C., Duboue E. R. (2019). An adult brain atlas reveals broad neuroanatomical changes in independently evolved populations of Mexican cavefish. Frontiers in Neuroanatomy, 13, 88. 10.3389/fnana.2019.00088 31636546 PMC6788135

[bib34] Losos J. B., Jackman T. R., Larson A., Queiroz K. d., Rodríguez-Schettino L. (1998). Contingency and determinism in replicated adaptive radiations of island lizards. Science, 279(5359), 2115–2118. 10.1126/science.279.5359.2115 9516114

[bib35] Maan M. E., Seehausen O., Groothuis T. G. (2017). Differential survival between visual environments supports a role of divergent sensory drive in cichlid fish speciation. The American Naturalist, 189(1), 78–85. 10.1086/689605 28035885

[bib37] McMillan W. O., Jiggins C. D., Mallet J. (1997). What initiates speciation in passion-vine butterflies?. Proceedings of the National Academy of Sciences, 94(16), 8628–8633. 10.1073/pnas.94.16.8628 PMC23051 9238028

[bib36] Mallet J., McMillan W. O., Jiggins C. D. (1998). Estimating the mating behavior of a pair of hybridizing *Heliconius* species in the wild. Evolution; International Journal of Organic Evolution, 52(2), 503–510. 10.1111/j.1558-5646.1998.tb01649.x 28568341

[bib38] Merrill R. M., Chia A., Nadeau N. J. (2014). Divergent warning patterns contribute to assortative mating between incipient *Heliconius* species. Ecology and Evolution, 4(7), 911–917. 10.1002/ece3.996 24772270 PMC3997309

[bib39] Montgomery S. H., Merrill R. (2017). Divergence in brain composition during the early stages of ecological specialization in *Heliconius* butterflies. Journal of Evolutionary Biology, 30(3), 571–582. 10.1111/jeb.13027 27981714

[bib40] Montgomery S. H., Merrill R. M., Ott S. R. (2016). Brain composition in *Heliconius* butterflies, posteclosion growth and experience-dependent neuropil plasticity. Journal of Comparative Neurology, 524(9), 1747–1769. 10.1002/cne.23993 26918905

[bib41] Montgomery S. H., Mundy N. I., Barton R. A. (2016). Brain evolution and development: Adaptation, allometry and constraint. Proceedings of the Royal Society B: Biological Sciences, 283(1838), Article 20160433. 10.1098/rspb.2016.0433 PMC5031648 27629025

[bib42] Montgomery S. H., Ott S. R. (2015). Brain composition in *Godyris zavaleta*, a diurnal butterfly, reflects an increased reliance on olfactory information. Journal of Comparative Neurology, 523(6), 869–891. 10.1002/cne.23711 25400217 PMC4354442

[bib43] Montgomery S. H., Rossi M., McMillan W. O., Merrill R. M. (2021). Neural divergence and hybrid disruption between ecologically isolated *Heliconius* butterflies. Proceedings of the National Academy of Sciences, 118(6), Article e2015102118. 10.1073/pnas.2015102118 PMC8017967 33547240

[bib44] Moore J. S., Hendry A. P. (2005). Both selection and gene flow are necessary to explain adaptive divergence: Evidence from clinal variation in stream stickleback [Article]. Evolutionary Ecology Research, 7(6), 871–886. https://www.scopus.com/inward/record.uri?eid=2-s2.0-26444585641&partnerID=40&md5=1c458eeffcbbe63d12410051f14b185a

[bib45] Morante J., Desplan C. (2004). Building a projection map for photoreceptor neurons in the *Drosophila* optic lobes. Seminars in Cell & Developmental Biology, 15, 137–143. 15036216 10.1016/j.semcdb.2003.09.007

[bib46] Morris B. J., Couto A., Aydin A., Montgomery S. H. (2021). Re-emergence and diversification of a specialized antennal lobe morphology in ithomiine butterflies. Evolution; International Journal of Organic Evolution, 75(12), 3191–3202. 10.1111/evo.14324 34383301

[bib47] Muñoz A. G., Salazar C., Castano J., Jiggins C., Linares M. (2010). Multiple sources of reproductive isolation in a bimodal butterfly hybrid zone. Journal of Evolutionary Biology, 23(6), 1312–1320. 10.1111/j.1420-9101.2010.02001.x 20456567

[bib48] Niven J. E., Laughlin S. B. (2008). Energy limitation as a selective pressure on the evolution of sensory systems. Journal of Experimental Biology, 211(11), 1792–1804. 10.1242/jeb.017574 18490395

[bib49] Nosil P., Crespi B. J., Sandoval C. P. (2002). Host-plant adaptation drives the parallel evolution of reproductive isolation [Article]. Nature, 417(6887), 440–443. 10.1038/417440a 12024213

[bib50] Nosil P., Vines T. H., Funk D. J. (2005). Perspective: Reproductive isolation caused by natural selection against immigrants from divergent habitats. Evolution; International Journal of Organic Evolution, 59(4), 705–719. http://www.jstor.org/stable/3449020, Date accessed January, 2025. 15926683

[bib51] Oke K. B., Rolshausen G., LeBlond C., Hendry A. P. (2017). How parallel is parallel evolution? A comparative analysis in fishes [Article]. The American Naturalist, 190(1), 1–16. 10.1086/691989 28617637

[bib52] Ott S. R. (2008). Confocal microscopy in large insect brains: Zinc–formaldehyde fixation improves synapsin immunostaining and preservation of morphology in whole-mounts. Journal of Neuroscience Methods, 172(2), 220–230. 10.1016/j.jneumeth.2008.04.031 18585788

[bib53] Paulk A. C., Dacks A. M., Phillips-Portillo J., Fellous J.-M., Gronenberg W. (2009). Visual processing in the central bee brain. The Journal of Neuroscience, 29(32), 9987–9999. 10.1523/JNEUROSCI.1325-09.2009 19675233 PMC2746979

[bib54] Prada C., Hellberg M. E. (2014). Strong natural selection on juveniles maintains a narrow adult hybrid zone in a broadcast spawner. The American Naturalist, 184(6), 702–713. 10.1086/678403 25438171

[bib55] Rivas-Sánchez D. F., Gantiva-Q C. H., Pardo-Díaz C., Salazar C., Montgomery S. H., Merrill R. M. (2024). Parallel shifts in flight-height associated with altitude across incipient *Heliconius* species. Journal of Evolutionary Biology, 37(1), 123–129. 10.1093/jeb/voad003 38285663

[bib56] Rivas-Sánchez D. F., Melo-Flórez L., Aragón A., Pardo-Díaz C., Salazar C., Montgomery S. H., Merrill R. M. (2023). Parallel evolution of behavior, physiology, and life history associated with altitudinal shifts in forest type in *Heliconius* butterflies. Evolution; International Journal of Organic Evolution, 77(6), 1458–1467. 10.1093/evolut/qpad062 37075171

[bib57] Rundle H. D., Nosil P. (2005). Ecological speciation. Ecology Letters, 8(3), 336–352. 10.1111/j.1461-0248.2004.00715.x

[bib58] Schluter D. (2000). The ecology of adaptive radiation. Oxford University Press.

[bib59] Schluter D. (2009). Evidence for ecological speciation and its alternative. Science, 323(5915), 737–741. 10.1126/science.1160006 19197053

[bib60] Seymoure B. M., Mcmillan W. O., Rutowski R. (2015). Peripheral eye dimensions in longwing (*Heliconius*) butterflies vary with body size and sex but not light environment nor mimicry ring. The Journal of Research on the Lepidoptera, 48, 83–92. 10.5962/p.266475

[bib61] Spitze K. (1993). Population structure in *Daphnia obtusa*: Quantitative genetic and allozymic variation. Genetics, 135(2), 367–374. 10.1093/genetics/135.2.367 8244001 PMC1205642

[bib62] Stöckl A., Heinze S., Charalabidis A., el Jundi B., Warrant E., Kelber A. (2016). Differential investment in visual and olfactory brain areas reflects behavioural choices in hawk moths. Scientific Reports, 6(1), 26041. 10.1038/srep26041 27185464 PMC4869021

[bib63] Stuart Y. E., Veen T., Weber J. N., Hanson D., Ravinet M., Lohman B. K., Thompson C. J., Tasneem T., Doggett A., Izen R. (2017). Contrasting effects of environment and genetics generate a continuum of parallel evolution. Nature Ecology & Evolution, 1(6), 1–7. 28812631 10.1038/s41559-017-0158

[bib64] Tan S., Amos W., Laughlin S. B. (2005). Captivity selects for smaller eyes. Current Biology, 15(14), R540–R542. 10.1016/j.cub.2005.07.019 16051157

[bib65] Van Belleghem S. M., Cole J. M., Montejo-Kovacevich G., Bacquet C. N., McMillan W. O., Papa R., Counterman B. A. (2021). Selection and isolation define a heterogeneous divergence landscape between hybridizing *Heliconius* butterflies. Evolution; International Journal of Organic Evolution, 75(9), 2251–2268. 10.1111/evo.14272 34019308 PMC8454027

[bib66] Van Belleghem S. M., Rastas P., Papanicolaou A., Martin S. H., Arias C. F., Supple M. A., Hanly J. J., Mallet J., Lewis J. J., Hines H. M., Ruiz M., Salazar C., Linares M., Moreira G. R. P., Jiggins C. D., Counterman B. A., McMillan W. O., Papa R. (2017). Complex modular architecture around a simple toolkit of wing pattern genes. Nature Ecology & Evolution, 1(3), 0052. 10.1038/s41559-016-0052 28523290 PMC5432014

[bib67] van Woerden J. T., van Schaik C. P., Isler K. (2010). Effects of seasonality on brain size evolution: Evidence from Strepsirrhine primates. The American Naturalist, 176(6), 758–767. 10.1086/657045 21043783

[bib68] Via S. (2009). Natural selection in action during speciation. Proceedings of the National Academy of Sciences, 106(supplement_1), 9939–9946. 10.1073/pnas.0901397106 PMC2702801 19528641

[bib69] Warton D. I., Duursma R. A., Falster D. S., Taskinen S. (2012). smatr 3—An R package for estimation and inference about allometric lines. Methods in Ecology and Evolution, 3(2), 257–259. 10.1111/j.2041-210X.2011.00153.x

[bib70] Wcislo W. T. (1989). Behavioral environments and evolutionary change. Annual Review of Ecology and Systematics, 20(1), 137–169. 10.1146/annurev.es.20.110189.001033

[bib71] Wright D. S., Rodriguez-Fuentes J., Ammer L., Darragh K., Kuo C.-Y., McMillan W. O., Jiggins C. D., Montgomery S. H., Merrill R. M. (2023). Adaptive divergence in the eyes of *Heliconius* butterflies likely contributes to pre- and post mating isolation. bioRxiv 564160. 10.1101/2023.10.26.564160, October 31, 2023, preprint: not peer reviewed.

[bib72] Yuan F., Bernard G. D., Le J., Briscoe A. D. (2010). Contrasting modes of evolution of the visual pigments in *Heliconius* butterflies. Molecular Biology and Evolution, 27(10), 2392–2405. 10.1093/molbev/msq124 20478921

