# Supplementary material for: Repeated evolution of reduced visual investment at the onset of ecological speciation in high-altitude Heliconius butterflies
Source: Evol Lett. 2025 Jul 9;9(4):502–10. doi: 10.1093/evlett/qraf017 (PMC12448208; doi:10.1093/evlett/qraf017)
Supplement: qraf017_Supplemental_Files [file qraf017_supplemental_files.zip › Supplementary material 1 (final version).docx]

**Supplementary methods**

We obtained georreferenced occurrance data (Rosser et al., 2012) for Nymphalid butterflies associated with the Heliconiini adaptive radiation (Cicconardi et al., 2023). These records were filtered for outliers and spatially thinned to reduce autocorrelation and sampling bias using the *thin* function in the *spThin* R package (Aiello‐Lammens et al., 2015). For each georeferrenced point, we extracted altitude measurements from a publicly available database (Fick & Hijmans, 2017), which allowed us to estimate the median altitude at the range of each species. We chose the median rather than the mean altitude in our analyses because it is less sensitive to extreme values and thus may more accurately represent the altitude conditions experienced by the majority of individuals of a given specie.

We sorted our species following Cicconardi et al. (2023)’s phylogenetic tree. Using the *fit* function of the R package *Phytools* (Revell, 2012), we estimated altitude at the internal nodes of the tree, which represent ancestral states of descendant branches. We focused on the nodes representing ancestors of:

1. the Erato group (which contains *Heliconius erato* subspecies as well as the closely related *H. himera*, and more distantly related species such as *H. hermathena*, *H. hecalesia*, *H. clysonymus* and *H. telesiphe*),
2. the *H. erato* subspecies (and the closely related *H. himera* and *H. chestertonii*), also referred to as the *erato* species complex
3. the species pair *H. himera*-*H. e. lativitta,* noting that the remaining populations included in our main analysis were not available due to their absence from the phylogenetic tree.
4. the high altitude *H. telesiphe*, *H. clysonymus* and *H. hortense*, which form a clade of high altitude specialists that are independent of the *erato* species complex, but which raise the possibility that the last common ancestor of the Erato group was also a high altitude population, with some descendent lineages secondarily returning to low altitude forest.

We then compared the altitude at these nodes with the median altitude of extant species (supplementary table 1).

**Supplementary results and discussion**

The extant nymphalid species used in the phylogenetic tree (supplementary figure 1) range in median altitude from 39 to 2500 m. Our ancestral reconstruction adscribes:

1. a 664.98 (node 98, 95% CI [291.77, 1038.19]) m altitude to the ancestor of the Erato group,
2. a 503.27 (node 103, 95% CI [196.39, 810.15]) m elevation to the ancestor of the *erato* species complex
3. a 657.78 (node 106, 95% CI [353.44, 962.13]) m altitude to the ancestor of *Heliconius erato lattivita* and *Heliconius himera.*
4. a 1039.12 (node 99, 95% CI [630.52, 1447.73]) m elevation to the ancestor of the high-altitude species *Heliconius clysonymus*, *Heliconius telesyphe* and *Heliconius hortense*.

These results are visually represented in the supplementary figure 1 and described in more detail in the accompanying supplementary table.

Ancestral state reconstructions suggest that the ancestor from which taxa of the Erato group emerged had a low altitudinal range, comparable to the majority of extant species (supplementary figure 1, and accompanying table). Within the Erato group, “high-altitude” ranges evolved in the branch that gave rise to the *H. clysonymus-H. telesyphe* and *H. hortense* clade, indicating that these closely related species share this trait by common descent. In contrast, the node representing the ancestor of the extant *erato* species complex, including all *erato* subspecies, *H. himera* and *H. chestertonii* (although not included in the tree) is associated with a low-altitude range, despite *H. himera* having a high-altitude range. An implication is therefore that high-altitude ranges have independently evolved from low-altitude populations within the Erato group more than once. Although the phylogeny we used does not include *H. chestertonii* in our analyses, this species is clustered separately from *H. himera* in the most recent phylogeny of these taxa (Van Belleghem et al., 2021), suggesting an additional independent shift towards high-altitude. *H. himera* and *H. chestertonii* are the only high-altitude species of the *erato* complex (Arias et al., 2008; Jiggins et al., 1996). While it could be argued that the inclusion of *H. chestertonii* may increase the estimated range for the *erato* species complex ancestor, more than a dozen low altitude *erato* races are also not included in our tree (Rosser et al., 2012) and we therefore believe this effect can be discounted. The high altitude ranges of *H. himera* and *H. chestertonii* are therefore most likely derived relative to lower altitude taxa such as *H. e. cyrbia* and *H. e. venus*, which would resemble the ancestor of the *erato* complex more closely.

Supplementary figure 1. Time-calibrated phylogenetic relationships between nymphalid butterflies (modified from (Cicconardi et al., 2023)).The colour scale conveys altitude and relevant nodes are labeled with numbers. The tree and the ancestral reconstruction estimates were created using the R package Phytools (Revell, 2012). Node altitude estimates and confidence intervals provided in the acompanying table below.

Accompanying table of Supplementary figure 1.

| Ancestral state estimates | | | | |
| --- | --- | --- | --- | --- |
| **Node** | **Ancestor of** | **Estimated altitude (m)** | **Lower confidence interval** | **Higher confidence interval** |
| 98 | *Erato* group | 664.98 | 291.77 | 1038.19 |
| 99 | *H. clysonymus* + *H. telesyphe* + *H. hortense* | 1039.12 | 630.52 | 1447.73 |
| 101 | *Erato* complex + *H. hecalesia* + *H. hermathena* | 612.77 | 226.86 | 998.67 |
| 102 | *Erato* complex + *H. hermathena* | 494.51 | 113.58 | 875.45 |
| 103 | *Heliconius erato* (*Erato* complex) | 503.27 | 196.39 | 810.15 |
| 106 | *H. e. lattivita* - *H. himera* | 657.78 | 353.44 | 962.13 |

**Supplementary References**

Aiello‐Lammens, M. E., Boria, R. A., Radosavljevic, A., Vilela, B., & Anderson, R. P. (2015). spThin: an R package for spatial thinning of species occurrence records for use in ecological niche models. *Ecography*, *38*(5), 541-545.

Arias, C. F., Munoz, A. G., Jiggins, C. D., Mavarez, J., Bermingham, E., & Linares, M. (2008). A hybrid zone provides evidence for incipient ecological speciation in Heliconius butterflies. *Molecular Ecology*, *17*(21), 4699-4712.

Cicconardi, F., Milanetti, E., Pinheiro de Castro, E. C., Mazo-Vargas, A., Van Belleghem, S. M., Ruggieri, A. A., Rastas, P., Hanly, J., Evans, E., Jiggins, C. D., Owen McMillan, W., Papa, R., Di Marino, D., Martin, A., & Montgomery, S. H. (2023). Evolutionary dynamics of genome size and content during the adaptive radiation of Heliconiini butterflies. *Nat Commun*, *14*(1), 5620. <https://doi.org/10.1038/s41467-023-41412-5>

Fick, S. E., & Hijmans, R. J. (2017). WorldClim 2: new 1‐km spatial resolution climate surfaces for global land areas. *International journal of climatology*, *37*(12), 4302-4315.

Jiggins, C. D., McMillan, W. O., Neukirchen, W., & Mallet, J. (1996). What can hybrid zones tell us about speciation? The case of Heliconius erato and H. himera (Lepidoptera: Nymphalidae). *Biological Journal of the Linnean Society*, *59*(3), 221-242.

Revell, L. J. (2012). phytools: an R package for phylogenetic comparative biology (and other things). *Methods in Ecology and Evolution*(2), 217-223.

Rosser, N., Phillimore, A. B., Huertas, B., Willmott, K. R., & Mallet, J. (2012). Testing historical explanations for gradients in species richness in heliconiine butterflies of tropical America. *Biological Journal of the Linnean Society*, *105*(3), 479-497.

Van Belleghem, S. M., Cole, J. M., Montejo-Kovacevich, G., Bacquet, C. N., McMillan, W. O., Papa, R., & Counterman, B. A. (2021). Selection and isolation define a heterogeneous divergence landscape between hybridizing Heliconius butterflies. *Evolution*, *75*(9), 2251-2268. <https://doi.org/https://doi.org/10.1111/evo.14272>
